# Etiology, consequences, and solutions of working women’s work-life conflict: a qualitative study

**DOI:** 10.1186/s12905-023-02873-4

**Published:** 2024-01-23

**Authors:** Zahra Hosseini, Seyyede Fateme Rahimi, Fatemeh Salmani, Mohammad Reza Miri, Teamur Aghamolaei, Reza Dastjerdi

**Affiliations:** 1 Social Determinants in Health Promotion Research Center, Hormozgan Health Institute, Hormozgan University of Medical Sciences, Bandar Abbas, Iran; 2 Student Research Committee, Hormozgan University of Medical Sciences, Bandar abbas, Iran; 3 Department of Epidemiology and Biostatistics, School of Health, Social Determinants of Health Research Center, Birjand University of Medical Sciences, Birjand, Iran; 4 Department of Health Promotion and Education, School of Health, Social Determinants of Health Research Center, Birjand University of Medical Sciences, Birjand, Iran; 5 Department of Health Promotion and Education, School of Health, Social Determinants in Health Promotion Research Center, Research Institute for Health, Hormozgan University of Medical Sciences, Bandar Abbas, Iran; 6 Department of General Courses, School of Medicine, Cardiovascular Diseases Research Center, Birjand University of Medical Sciences, Birjand, Iran

**Keywords:** Work-life conflict, Women, Employee, Organization, Work-life balance

## Abstract

**Introduction:**

Work-life conflict (WLC) is important in organizational behavior research and human resource management. The present research aimed to investigate the underlying causes, consequences, and solutions to WLC in Iranian working women.

**Materials and methods:**

The present qualitative study was conducted through a content analysis method among 19 working married women in Birjand, a city in the east of Iran, from December 2021 to February 2022. To collect the data, semi-structured interviews were held. The average interview time was 45 minutes, and all interviews were recorded upon the participants’ consent. Finally, after coding, the information was analyzed with MAXQDA software.

**Findings:**

The causes of conflict included 4 main categories of individual, interpersonal, organizational, and cultural factors, with seven subcategories: the pressure of the mother’s role at home, personality traits, lack of individual skills, insufficient support, work characteristics, organizational policies, and the traditional role of women in society.

The consequences of conflict included 2 main categories, Decreasing quality of life and work problems with 4 subcategories: physical and mental illnesses, forgetting one’s role towards others, limiting social communication, and reducing productivity.

Conflict resolution methods included 3 main classes of individual-oriented, other-oriented, and organization-oriented with 8 subclasses: program-oriented, meaning-oriented, emotion-oriented, avoidance, emotional support, instrumental and work support, support work policies, and correct management views.

**Conclusion:**

To solve the problem of conflict, different aspects should be considered and help to solve this challenge by influencing each dimension.

**Supplementary Information:**

The online version contains supplementary material available at 10.1186/s12905-023-02873-4.

## Introduction

The emergence and development of families where spouses are both employed and raise income is a global social phenomenon [1]. In today’s modern work environment, maintaining work-life balance (WLB) is essential for employees because they are expected to perform multiple tasks simultaneously and have maximum productivity [2]. Work-life balance is a concept that is poorly and variably defined, and perhaps the difficulty in defining this concept reflects its complexity [3], Kirchmeyer (2000) defined work–life balance as “achieving satisfying experiences in all life domains and to do so requires personal resources such as energy, time, and commitment to be well distributed across domains” [4]. Actually, WLB is the required coordination of an employee’s professional and personal life [5]. It is difficult to strike a balance in modern societies in response to the increasing demands of family and work. Because employees must assume different roles in both conditions [6].

Work- life conflict (WLC) has become an important issue in investigating organizational behavior and human resource management, which is a positive correlate of employees’ work-life health [7] and organizational performance [8]. Mismanagement and inconsistency in life, family, and work as a result WLC can damage the individuals’ lives, organizations, and even society [9, 10]. Although men also face WLC, women are more affected because they have to take care of family and children and do the domestic chores [11]. Studies have shown that women are always more stressed than men, and in particular, full-time women whose children are less than 13 years old are the most conflicted [12], because they usually fail to spend sufficient time with children. As a result, they are forced to leave work in some cases. Therefore, they face the increased pressure of professional and personal life, which causes role conflict and stress [9] at work and in life [13]. In addition, WLC is associated with reduced job satisfaction, absenteeism, lower performance, and intention to leave the organization [14]. In the twenty-first century, Women are working in all manufacturing and service sectors throughout the world [9]. Therefore, it is necessary for working women to achieve work-life balance (WLB). To this aim, they have to carefully handle their personal balance and skillfully blend their roles so as to optimize their potential in all aspects of life [15].

Studies show that work-life conflict has adverse effects on family and workplace, and generally endangers the well-being of societies [2, 16]. People who experience work and life conflict have less productivity and commitment, and are more likely to be absent or leave the organization [16, 17]. In the family dimension, work-life conflict can cause bad mood and misbehavior with spouse and children, inadequate and inappropriate performance of parental and marital duties, and ultimately decrease life satisfaction and quality of life [15, 16]. In the individual dimension, people who have conflicts between work and life suffer significantly from a decrease in mental well-being and physical health [17, 18].

The prevalence of work-life conflict among women of different societies and cultures is different [15]. In Iran, a working woman faces more problems than a working man to strike a balance between work and life [19]. This research is basically designed as a qualitative study to better understand local and cultural differences that affect work-life conflict. Thus, the main purpose is to answer the following questions as perceived by working women in universities of Birjand (in the east of Iran):

- What are the causes of work-life conflict in working women?
- What are the consequences of work-life conflict in personal and professional lives of working women?
- As perceived by working women, what solutions are there to solve the work-life conflict?

Considering the importance of work-life conflict for the lives of individuals, organizations and even society, a qualitative we can provide a deeper understanding of the feelings and concerns of female employees with work-life conflicts and offer solutions to create a WLB.

## Literature review

According to the gender role theory, because women are more exposed to work-life conflict even when employed, the traditional duties of housekeeping and care-taking of family members are expected from them [2, 18]. This situation is more tangible in eastern societies, which are more traditional. In this regard, several studies have investigated the causes and effects of work and life conflict or work and family conflict in working women around the world using different statistical methods. As the findings showed, it is possible to understand the commonalities and differences of the causes of work-life conflict in working women across different cultures. In the following, we will provide a brief description of the results of recent studies:

In a qualitative study, Taghizadeh et al. (2021) concluded that some men in traditional cultures expect women to always be subservient, which is widely known as hegemonic masculinity. In addition to this job stress, the high load of family duties and lack of individual abilities and skills are considered other causes of conflict [19]. In explaining these results, especially the category of hegemonic masculinity, mention can be made of Rafiq et al.’s study (2023), showing that authoritarian leadership is positively correlated with work-family conflict and negatively with emotional exhaustion [20]. Gender perspectives on women’s employment and type of job may even affect women’s job choice. For example, the findings some qualitative research by Hafeez et al. conducted on Pakistani women entrepreneurs showed that women’s choice to become entrepreneurs is at three levels: internal, external and interpersonal. Also, the results showed that gender roles and gender-related job segregation play an important role and influence women’s job choice [21]. Tasnim et al. (2017) in Bangladesh found that long working hours, hard work, excessive workload, childcare responsibilities, workplace discrimination and prejudice, lack of supervision and dominant management style, and lack of family support are the underlying reasons for disrupted maintenance of work and life balance in working women [14]. Ademuyiwa et al. (2022) in Nigeria investigated the consequences of work-life conflict in working women and found that stress, mental fatigue and burnout as psychological disorders are among the major effects of this challenge. Also, as their results showed, closing work at the right time, helping with housework, having a kindergarten near work, and getting help from colleagues at work were mechanisms that facilitate and reduce conflict [22].

## Materials and methods

The present qualitative study used a content analysis in Birjand, a city in the east of Iran. It was conducted from December 2021 to February 2022.

### Participants

The participants were women working at Birjand University of Medical Sciences. The working environment is another variable that may influence the level of work-life balance.

The university is an arena prone to work and life conflict. Working women in universities experience conflict and role accumulation widely [23].

Particularly those who work in institutions with intense work stress are more challenged to assure work-life balance. Among those intensively faced with this challenge are university employees [2].

To enrich the data, the managers and spouses of some of these women were also included in the study The sampling was purposive with a maximum variation of education, work experience, type of job, place of working and family size. Since husbands spend a large part of their time with their wives, these experiences could also help understand the issue. Interviewing the husbands of working women and university managers is actually a form of triangulation to acquire a more comprehensive understanding of the subject.

### Inclusion and exclusion criteria

The inclusion criteria were femininity, being married, working at university, having a work-life conflict issue, at least 1 year of work experience, and an informed consent to participate in the study. To identify women with work-life conflicts, 320 women completed the Carlson standard work-life conflict questionnaire, the score range of which was 18–90. A higher score indicated more conflict. The research team considered a score above 40 as the cut-off point for entering the study.

The minimum and maximum score of Carlson’s standard questionnaire is 18 and 90. The first 33% of the conflict score range between 18 and 42 (interpreted as low conflict). The second 33% of scores ranged between 43 and 66 (interpreted as moderate conflict). The third 33% of scores were between 67 and 90 (interpreted as high conflict). Therefore, the range of moderate to high conflict was decided to be the score of 40, to be on the safe side, as the inclusion criterion for the interview. The lowest conflict score of the interviewed person was 43 (Maryam, 35 years old).

The exclusion criterion was the participant’s unwillingness to continue the interview. If a participant was interviewed for less than 30 minutes, or if the researcher decided that the participant answered the questions conservatively and did not want to overtly state his experiences, the participant was excluded from the study and another eligible person was replaced.

### Ethical considerations

This study was approved by an Ethics Committee of Research and the deputy of research at Hormozgan University of Medical Sciences (# IR.HUMS.REC.1400.214), and received a clinical trial code (#IRCT20210918052508N1). Written informed consent was obtained from all participants (320 women in the work-life conflict investigation stage, 19 women who were selected for interviews, and 6 women’s husbands in the qualitative stage) and it was explained to them that the information obtained from the questionnaires and the interviews (audio and written) will remain completely confidential in all stages of the research and the results will be published anonymously. Why the audio recorder was used was explained to the participants and the interviews were recorded with their permission. All the steps performed were according to the ethical code of the Declaration of Helsinki. Also, the principle of confidentiality was observed for the information obtained from the questionnaires of the first stage of assessing work-life conflict and the audio content of the people who were excluded from the study for any reason, and the information obtained from these people was not included in the results presented in this article, such as the information obtained. The final participants in this research have been kept confidential with the research team.

### Data collection and analysis

Semi-structured in-depth interviews were used to collect the data. A total number of 29 interviews were held with working women, 4 with managers and 6 with women’s husbands. Each interview lasted between 30 and 45 minutes.

It was arranged to have a separate room for interviewing participants in Birjand University of Medical Sciences. The place was supposed to be somewhere that women could talk comfortably without interference. If the participant did not want or did not have the time to interview us during working hours, the interview was held in any place that he suggested outside the workplace (participant’s home or public places in the town). The interviews were held one on one and only in the presence of researcher to preserve the privacy and comfort of the participants.

At the beginning of each session, after introducing himself and explaining the purpose of the study, the researcher enquired about the participants’ demographic information, including age, education level, employment status, number of children, etc. Interviewees were free to withdraw from the interview any time they wanted. The interview was voice-recorded. During the interview, notes were taken whenever necessary.

The interview began with these general questions:

- Tell about your job and life experiences.
- Tell about the conflict that happened between your job and life.

Then according to what the participants already provided; other questions were asked. Also, when needed, exploratory probes were added, such as “Please explain more”, “Please provide an example”, “What do you mean?”, “How did you feel about this?”

The participants’ moods and emotions were recorded too, such as a nervous laughter or hatred during the conversation, to understand and interpret the interview more accurately.

Immediately after the interviews, they were first transcribed on paper and were then typed.

MAXQDA can deal with different data formats and can support data in Persian language. It can easily manage and organize the content of interviews and perform accurate qualitative analyses at a high speed and ease. The present data were analyzed in MAXQDA 2020 [24].

Having extracted the initial codes, the conceptually similar or semantically related items were categorized. This process of analysis continued until the main categories and subcategories were obtained.

### Methodological considerations

Acceptance, validity, confirmability, and transferability criteria were used to determine the accuracy of data [16]. In this study, the researcher first wrote down his personal ideas, values, and judgments and how they could affect the data collection and analysis. He recorded his thoughts about the answers he expected to hear from the participants and tried to avoid biasing the results.

When we are not sure of heterogeneity of experience, sample selection becomes further demanding. An inappropriate strategy can misrepresent the population and lead to the failure of future plans and policies. Sampling strategy, if properly planned, provides the first line of defense against errors and biases in collecting sample data in a population with heterogeneous experiences. We used the word varimax for this purpose [11]. That is, we tried to observe the maximum variety in selecting the sample in terms of age, length of employment, type of employment, number of children, age of the youngest child, etc.

The trustworthiness of qualitative research corresponds to the validity and reliability of quantitative research. It was tested Guba and Lincoln’s criteria [25]. Credibility can be achieved through the researcher’s long-term engagement with information, and appropriate interaction with participants. Dependability includes activities that increase the accuracy of the generated data. In the present research, trustworthiness was provided by transcribing the recorded interviews as soon as possible and re-reading the entire data. Confirmability is ensured by observing the impartiality of the researcher and using the review of observers and agreement on the codes and themes by the team of researchers. Transferability is the generalizability of findings to similar situations. In the present study, to check transferability, maximum variety was used in sampling.

The researcher was constantly immersed in the data, shared the coding and text with the participants, rechecked the codes by other researchers and used a varimax sampling of participants in terms of age groups, work areas, family size, husband’s job and so on to increase the validity of findings.

The impression that was made from the interviewee’s words was repeated for the same participant during the interview to confirm the accuracy of interpretation.

First, 320 women completed the standard Carlson WLC questionnaire, of which 57% (*n* = 182) had WLC. Among these women, 19 were interviewed purposively. The results showed that most of them had a bachelor’s degree, 1–2 children, 41–50 hours of work per week, and a conflict score of 40–50, permanent employment and 10–15 years of experience (Table 1).

**Table 1:** The research participants’ demographic information

Variable	Dimension	Frequency	Percentage
Education level	diploma	2	10.52
	Associate degree	4	21.05
	Bachelor’s degree	6	31.59
	Master’s degree	5	26.32
	Doctorate	2	10.52
Number of children	0	4	21.05
	1–2	11	57.90
	≤3	4	21.05
Age of the youngest child	< 2 years	5	26.32
	2–5 years	4	21.05
	5–8 years	6	31.58
	> 8 years	4	21.05
Working hours per week	≤40	6	31.58
	41–50	9	47.37
	≥51	4	21.05
Conflict score	40–50	12	63.16
	51–60	7	36.84
Type of employment	permanent	7	36.84
	fixed term	4	21.05
	contractual	3	15.79
	human resource project	5	26.32
Work experience	< 5 years	3	15.79
	5–10 years	4	21.05
	10–15 years	6	31.58
	15–20 years	2	10.52
	20–25 years	3	15.79
	> 25 years	1	5.27
Age	25–30 years	5	26.32
	31–35 years	3	15.79
	36–40 years	5	26.31
	41–45 years	3	15.79
	> 45 years	3	15.79

Moreover, six of the women’s husbands were interviewed, whose ages ranged from 39 to 50 years. In addition, four managers, including 2 Ph.D. holders and two master degree holders were interviewed. All had more than 25 years of work experience (range = 25–33) and 47–62 years of age.

In the data analysis process, the codes were checked 5 times (once by the interviewer, 2 times by the research team and 2 times by an expert team outside the research) in order to increase the validity and accuracy of the data.

In the following, the categories and sub-categories are introduced. (Table 2).

**Table 2:** Categories and sub-categories of the analysis of interviewees’ experiences of WLC

Sub- subcategory	Sub-category	Main categories
Causes of conflict	personal factors	The pressure of a mother’s role at home
		Personality traits
		Lacking personal skill
	Interpersonal factors	Lack of emotional support
		Lack of instrumental and work support
	Organizational factors	Features of work
		Organizational policies
	Cultural factors	The traditional role of women in society
		Patriarchy
Consequences of conflict	Lowered quality of life	Physical and mental problems
		Limited social communication
		Forgetting the other roles
	Job problems	Decreased productivity
		Loss of credit or job status
Conflict resolution methods	Individual-centered solutions	Program oriented
		Meaning oriented
		emotional oriented
		Avoidance
	Alternative ways of working	Emotional and practical support
	Organization-oriented solutions	Conservative work policies
		Correct management views

## Causes of conflict

### Category of personal factors

#### The pressure of mother’s role at home

Due to the multiple motherly and occupational roles that the working women population have, they are mainly faced with an issue called work-motherhood conflict and role pressure. This condition has caused them to face many issues and challenges in family and work life.

“The mother’s role of is mostly at the end of the balance scale. As a rule, a mother is always thinking about her child. She is concerned with where the child is, what she is eating= and doing, which may disturb concentration at work”. [Maryam, 35 years old].

#### Personality traits

Personality is an important factor related to WLC. In Stavroula Leka’s study, personality factors alone accounted for 17% of the variance in the overall WLC levels [17]. By influencing coping strategies and accessing resources, personality traits increase the level of optimism and vitality and ultimately increase the psychological capacity. This increase, in turn, leads to a better management of work and family duties and a subsequent reduction in the conflict between the two.

For example, people who have more important protective factors against stress, such as self-control, self-esteem and high self-confidence and positive thinking, are less prone to role conflict between work and life [17, 26]. Some personality traits may cause consequences to be positively and negatively involved in challenges of a woman’s life, for example, idealistic women must bear more workload in the environment and at home because they only fully accept their way of working.

“I am an idealist. I have a set of standards in mind for my work. Now, whether in the office or at home, it is better to say I do not do something myself. I do not accept the quality of someone else’s work. This idealism makes me spend too much time on things. And sometimes I fall behind my daily plans”. [Shadi, 38 years old].

Also, people who face self-confidence issues or stress and anxiety experience a more difficult life and work. “If you are a stressful person and you do lack self-confidence, this will make you unable to focus on your work and life”. [Saeedah, 40 years old].

#### Lacking personal skills

At the core of work-life programs is empowerment. In these programs, the topics discussed are time management skills, effective management, decision-making and problem-solving skills. Among the topics raised, time management skills play an effective role in reducing work-family conflict [15].

“Actually, balance is important in all aspects of life, but it is more important for women, especially working women who take up many roles need to learn many skills for this job. They need to acquire communication skills when angry, life skills, problem solving skill, time management and mental pressure management. I recommend these because I am a psychologist myself”. [Reza, 52 years old].

Currently, for the employees of various departments in Iran, including employees working in universities of medical sciences, classes are held regularly to improve the quality of life in physical and mental dimensions and with the educational content of life skills, problem solving, planning and effective communication. Yet, these classes are not efficient enough. Among the reasons for the lack of efficiency, two cases can be exemplified. The first case is the low perceived importance to holding these classes by the employees. Many employees go home instead of attending classes! The second case is the unattractiveness of the classes held due to the lack of experienced professors and experts or holding of classes at the end of office hours when employees are mostly tired. It is necessary for the employees to be more attentive to holding these classes. In order to make the classes more efficient, they should expect the authorities to hold classes with maximum quality.

### Category of interpersonal factors

#### Lake of emotional support

Researchers believe that emotional support is next to social support, which is the expression of positive emotions, empathic understanding and encouraging the expression of feelings by the support recipient. Several measures can be taken including sympathy, attention, affection and interest towards the individual. Influenced by this type of support, people feel calm, confident and develop a sense of belonging to a place when stressed. On the other hand, emotional support promotes a sense of self-esteem that enables a person to accept and effectively deal with the situation [18].

“The support one receives from the husband as well as the interpersonal support are extremely important. When my husband and I sit together and think of solutions to solve a family problem, I have the feeling that my husband is also thinking about the same problem. For example, I decide to send my son to my mother’s house for a week, my husband suggested to send him to his uncle’s house. I liked it very much and now I am completely satisfied”. [Azam, 31 years old].

#### Lake of instrumental and work support

One of the first definitions of social support has been posed by Cobb (1976), who defines this concept to describe a condition when a person attaches value to him among the social networks of people who love him, and care for his well-being [19]. Social support at work is the extent to which people consider their well-being important through the resources within the work environment, including colleagues, managers or supervisors, and the wider organization in which they work [16]. This support can be in the form of emotional concern or empathy, informational and instrumental help provided by people such as colleagues, supervisors or family members [20]. The presence of support is a necessary asset to limit the adverse effects of stress and work-family conflict [21].

“A working woman must be supported; otherwise, she gets weakened. Especially the more children she has, the sooner she comes to a dead end. The support by her husband is very important. The husband’s cooperation, division of labor at home, support of those around her, including parents, or her husband, for example, taking children back from school”. [Morteza, 40 years old].

“I’m comfortable with some of my colleagues. Sometimes when I’m not feeling well, I ask others to help me, and this help reduces my work load and makes me feel better”. [Elaheh, 35 years old].

### Category of organizational factors

#### Features of work

Three semantic codes including: long working hours, role overload, and ambiguity of tasks were repeated a lot in the interviews, and the interviewers considered it as one of the work-related characteristics that plays an important role in creating conflict between work and life. The length of working hours per week is positively correlated with the work and life imbalance. Long working hours mean that working women have very little time for family. In almost all studies, long working hours lead to a stressful lifestyle and work-life imbalance [22]. Role overload means that employees are overworked both in their personal space and in their work environment [15], not only having an actual workload, but also the perception or belief that the workload and the multiplicity of responsibilities is too much for the individual. There is also the ambiguity of duties issue, which leads to the mismanagement of other roles and the increase in the WLC [23].

In some cases, due to the mismanagement in offices without consulting the employees or without considering living conditions and even the level of their skills and abilities, the employees are transferred to departments that are not in their specialized field or that require a lot of work. In these situations, employees may struggle with work-life conflict more than ever.

In some other cases, for reasons such as high workload, urgent and necessary plans, and other reasons, “overtime” may be imposed on employees without consulting them or without considering their conditions; Repeating this problem several times may make people have work-life conflict.

“There are so many job responsibilities that you can’t handle them all, so you get distressed. Once I was a designer, I was an expert in a department where I had to do everything myself. Sometimes I took the files home... My brain would sometimes stop working. My husband was a planner, so he helped me with the projects at home”. [Mojgan, 40 years old].

“Routine work with no inherent variety quickly bores us. When we get bored, this boredom is brought home. Finally, when we are tired at home, everything is negatively affected”. [Ali, 50 years old].

#### Organizational policies

The norms and values related to the nature of work, conceptualization of the ideal employee and employee relations, are known as the work culture that governs an organization [27]. Policies supporting WLB at the organizational level are more likely to focus on timing, for example longer holiday hours, part-time work alternatives, telecommuting or flexible working hours. Such general policies are approved and implemented in the organization with the aim of creating a sense of human resource control over working hours [24]. Policies supporting work and family are effective in reducing the WLC by creating incentives for human resources such as support packages and providing childcare facilities [28].

“Organizations should show a bit more flexibility towards female employees, and the manager should not expect a woman who is thinking about her family at home at 2 o’clock to stay in the office for longer hours of work”. [Razieh, 37 years old].

“Unfortunately, our organization does not consider any facilities for working mothers. Some organizations have a child care room. Well, this is very good, and makes it easy for people to work”. [Farzaneh, 44 years old].

### Category of cultural factors

#### The traditional role of women in society

An old saying in China contends that a husband is the breadwinner and a wife is the housewife. This proverb shows the clear division of gender roles in a traditional family [25]. Regarding the effect of gender on WLC, stereotypically, men work full-time outside home, while women take care of domestic and family chores. The gender-based division of roles is actually part of a collective culture that can affect the role pressure on women [29].

“In family, there are high and unreasonable expectations from women and mothers, as if the mother should do all the housework. Even the child has come to the same understanding that the mother should do the housework, and this really puts a lot of pressure on woman”. [Shima, 28 years old].

#### Patriarchy

Patriarchy is a social and ideological construct that considers men superior to women. Patriarchy is described as a social system in which men have authority over women, children, and property. Patriarchy encourages male leadership, male dominance and male power, and governs the view that housework is on a woman’s shoulders [26].

“Men also have the same traditional view of the past, a kind of patriarchy where cooking, sweeping, changing diapers, etc. are mother’s duties. How can a mother go to a great length and do all these things?” [Farzaneh, 44 years old].

## Consequences of conflict for women’s lives

### Low quality of life

#### Physical and mental diseases

Many studies show that WLC is a risk factor for people’s health [30–32] These effects can be psychological or physical. These consequences include an increased consumption of sedatives, stress, depression, and other mental disorders and psychosomatic symptoms such as sleep disorders, headaches, and fatigue [33].

“If I go ahead with this condition, my health is threatened. I understand that sometimes I get a fast heart rate or digestive problems. I also have sleep disorders”. [Maryam, 39 years old].

“Extremely tired from working a lot at home makes me unable to do my housework well. Sometimes, especially at night, I find it hard to concentrate and begin to feel dizzy. I also have this problem early in the morning because I lack sleep. My child wakes up several times at night for milk “. [Fateme, 27 years old].

#### Limited social communication

One important effect of the lacking WLB is the reduction of recreation and social interactions outside home. A conflicted person prefers to dedicate the hours she spends having fun with friends or parties to doing the backlog. This can cause a decrease in life satisfaction and a decrease in the psychological capacity of that individual [34].

“Many times, I bring the office work home, because it is not completed in the work time... Believe me, I don’t even find the time to call siblings”.

“Usually in the evenings, I do the housework and children’s work, and very often I refuse my friend’s invitation for a birthday party or a simple hang-out. I prefer to sit and do my own work”. [Maryam, 41 years old].

#### Forgetting the other roles

According to the role pressure theory, the overall system considers the individual’s role as a system which is excessively demanding; thus, the individual is not able to fully respond to all demands. Consequently, there are high chances that she faces a large load of irregular and conflicting role requirements. She may not be able to do the responsibilities and duties involved in each role completely and correctly.

“This high tension and fatigue make you forget some roles you already have and some duties each role entails. Sometimes two weeks may pass but I do not find the time to visit my sick mother, so I can only call her instead”. [Ilham, 45 years old].

“If you can’t bring balance back to your life, you will do the duties you have for children and husband... but incompletely, that means ineffectively”. [Mohammad Reza, 50 years old].

## Job problems

### Decreased productivity

The negative effects of employees’ WLC from an occupational perspective can include job dissatisfaction, lack of organizational commitment, job stress, and the intention to leave. All these, in turn, affect job performance, direct and indirect costs of absence from work, costs related to the loss and replacement of valuable employees, customer satisfaction, and organizational productivity [35].

“Too much work pressure makes a person lose temper, get moody and damage the relationship with one’s clients beyond expectations”. [Arezu, 39 years old].

“The issue of balance is a very important one, because if not resolved, it will gradually lead to job dissatisfaction and increase problems in the work environment. It can also create personal and family problems”. [Abbas, 47 years old].

### Loss of credit or job status

Evidently, every organization hopes to increase the efficiency and productivity of its output (goods/services), so the high-level performance of each employee is very important for the organization. On the contrary, studies show that WLC induces stress in employees and leads to organizational underachievement [36]. It is important for an organization to achieve an optimal level of performance from an employee, yet WLC limits such achievements. There is a negative correlation between employee performance and WLC. This issue leads to the degraded job position or the loss of occupational or organizational credibility [37].

“One of the assistants in our office is a woman. Sometimes she delayed because she had to take her children in the morning to drop them off at daycare or a relative’s house. In the evenings, if she was in the office, she could not go to pick her up. Her work project was transferred to the archive. And her child cried a lot”. [Akram, 35 years old].

## Conflict resolution methods

### Individual -centered solutions

#### Program-oriented

Psychologists who investigate stress distinguish between problem-focused coping strategies and emotion-focused coping strategies. For the former, actions can be taken to solve the problem. Problem-focused coping is a rational approach that attempts to change the conditions by changing something in the environment or the way a person interacts with the environment (Lazarus & Folkman, 1987). As described by the psychologists, problem-focused coping (taking responsibility strategies such as time management or eliminating stressors through problem solving) often promotes a sense of control by assuming that the current condition can be changed, stress can be reduced and the adverse effects can be reduced too [38].

“I have to plan and prioritize so that I can accomplish my work, I always put my life as a higher priority than work; I remind myself that it is not necessary to do all the housework in just one day”. [Atiyeh, 41 years old].

“I advise that the young make the division of labor a rule at home. If everyone learns to cooperate, the work will be done quickly and there will be no fatigue and trouble for the mother. What’s wrong with the man sweeping or doing the dishes? Washing or spreading out the baby clothes and the like?” [Hossein, 38 years old].

#### Emotional oriented

Emotion-related coping includes efforts to change an individual’s emotional response to a stressor [39]. Most researchers approach emotional coping as the most effective factor involved in the relationship between stress and illness. Avoidance coping style is known as an effective short-term strategy, but in the long run it hinders cognitive agreement and increases disability symptoms such as depression [40].

“Sometimes I lose control and may take out the problems of the workplace or the problems at home on my husband and children or shout at them. Yelling calms me down. [Fahima, 29 years old].

“Sometimes I get so tired that I cry out of necessity. Crying was a temporary relief from emotions and mental stress.” [Zahra, 41 years old].

#### Meaning oriented

Meaning-centered coping involves using one’s values, beliefs, and goals to reset life priorities, attaching positive value to ordinary events, and finding and remembering transcendental power. Meaning-centered coping in turn evokes positive emotions, which restore the existing resources [41].

“But you also have to trust and believe in yourself that you can handle things”. [Hamid Reza, 51 years old].

“I used to develop a headache in the evenings and get nervous for no reason, but when I followed a friend’s advice, I got a bunch of prayers. I prayed peacefully for half an hour in the evenings. It was very helpful”. [Mitra, 33 years old].

#### Avoidance

These methods involve the use of emotional and passive styles. But, as a result, the problem remains and these people do not actively deal with the problem, so there is always a level of anxiety induced by the existence of the problem. Frequent and indiscriminate use of avoidance strategies can increase people’s susceptibility to depression, anxiety and stress [42].

“Sometimes I become completely carefree and do nothing”. [Azam, 31 years old].

“Once in a while, he gets so tired, I tell him to take a few days off. Let’s go see our parents and take some time away from work”. [Mohammed, 29 years old].

## Alternative ways of working

### Having emotional and practical support

An investigation of the role of multiple social supports (workplace, supervisor, colleague, family) in WLB among Bangladeshi working women showed that all-round support (emotional and instrumental) can lead to a better WLB in women [43].

“Sometimes the opinions of colleagues with similar problems are very helpful. For example, a colleague said that she made two or three types of stew on Friday and froze them. For the work days of the week when she could have been busy and had hardly any time to cook, she quickly made rice and gave them to her family to eat”. [Fateme, 37 years old].

“Getting help from someone you trust is necessary. Now this help can act as some advice from someone who has the same problem as you. It can be like getting help with office work, when overworked, from someone who can help, or getting help from your father, mother and other family members. Evidently, the husband’s role is the most important”. [Qasem 60 years old].

## Organization-oriented solutions

### Conservative work policies

The perception of organizational support has a positive effect on WLB. This positive effect means that the better the employee’s perception of organizational support, the more balanced the employee’s work and family life [44]. Policies such as the possibility of teleworking [45], granting incentive leave [46] and providing welfare facilities such as a child care center [47] are very helpful in this regard.

“Now, I would like to express my opinion from an organizational perspective. Organizations and departments should, as far as possible, consider supporting facilities for working mothers, such as childcare allowances for working mothers with children under 6 years of age”. [Asia, 36 years old].

“Another point is that organizations should penalize employees who don’t use their paid holidays. Some people look at these holidays materialistically, but these holidays are key to striking balance, especially when you need to rest”. [Zahra, 41 years old].

“I wish there was a way for women to work from a distance, like during the Covid-19 pandemic, when we worked at home. There were no problems and we took better care of our families. Giving incentive leaves or leaves based on performance is a good idea too, but unfortunately these are not always available in all companies”. [Mojgan, 44 years old].

### Correct management views

Managers are significantly involved in the development of WLB policies and play a central role in turning WLB policies into practice and ensuring the existence of appropriate checks and balances in managing such practices [48]. Managers having awareness and a positive attitude towards the work and life balance issue among their employees can be effective in striking this balance in employees’ lives [49].

“Giving an incentive leave to a woman working well despite multiple roles should be applied in the policies of the organization and managers”. [Qasem, 60 years old].

“Managers should abandon the traditional view of the work-life relationship in which there is competition between work and life and should go for an approach which benefits the individual and the organization both in line with each other”. [Hamid Reza, 51 years old].

“A manager can be truly influential in the workplace. When you work in a place where there is a person on the top who does not understand the conditions of a working woman, there is a constant fear of being late or accomplishing a certain task late and being reprimanded for that which is stress-inducing”. [Saeeda, 31 years old].

## Discussion

Today, women’s active role in society is ever increasing, and in addition to its extensive economic benefits for organizations and societies, working women benefit from the personal advantages of this active role in life [13]. Yet, this active role has become challenging for women, and has created an imbalance between personal and professional life.

The novelty of this research is that in addition to working women, hysbands and university managers were also interviewed to get a more comprehensive view. Understanding the cause of conflicts and their solutions in terms of the three sides of the triangle (women, their husbands and managers) can help planners in health promotion and policymakers at higher organizational levels to achieve the benefits with correct planning to reduce conflict for the individual, her family and also the society.

In the present study, 4 main categories were extracted for the causes of conflict, including personal, interpersonal, organizational and cultural factors, with eight sub-categories of maternal role pressure at home, personality traits, lacking individual skills, insufficient support, work requirements, and organizational policies, the traditional role of women in society and patriarchy.

In the category of personal factors, one’s personality traits and lacking skills such as time management and the pressure of the motherly role were frequently mentioned. The relationship between personality traits and work-family conflict has been investigated by many researchers so far [50–52]. Personality is an important factor that can distinguish people from each other. Therefore, personality traits affect the way an individual behaves in different situations of life, job, education, marriage, etc. [53]. For example, highly sensitive and neurotic personalities tend to show negative reactions in stressful conditions such as anger, anxiety, depression, embarrassment, fear, guilt, aggression, impulsivity and sadness. A neurotic individual exerts less power in impulse control [17]. They are also less likely to cope with stressful demands in life and, therefore, when one area of life is in conflict with another, they are more likely to withdraw [54].

In another study, researchers investigated the relationship between role expectations in work and family and its contribution to conflict [55]. Similar to the present study, which considered work requirements such as lack of control over work or high work pressure as the cause of conflict, the aforementioned study concluded that high expectations of the work role and low individual control over work are associated with WLC. These researchers also found that low cooperation of family members leads to WLC, and social support at work is associated with reduced conflicts [55].

Another research conducted on 56 female police officers and 59 female teachers showed that a demanding job role and high expectations from the family role cause conflicts, and these conflicts prevail more in policewomen than female teachers [56].

In the interpersonal category of support, lack of emotional support and lack of instrumental support were the most prominent themes found in the content of interviews. Zimmet listed the sources of emotional support as family, friends, and significant others. In addition, in business life in the organizational context, the sources of social and emotional support are considered to be colleagues and managers [57]. Bourne and Wechsler, in their study, found the positive effect of social support on the employees’ health [58]. Besides, it is claimed that emotional support has a direct effect and an indirect effect (i.e., mediating effect) on job burnout [59]. According to koosek et al., receiving social support from managers and having work support can strike a balance between work and life [60]. Instrumental support refers to behaviors and attitudes of family members aimed at assisting day-to-day household activities, such as relieving the employee of household tasks or otherwise accommodating the employee’s work requirements. This allows the family member to focus his/her time and preserve energy for work when it might otherwise be scarce; suggesting that it positively influences the individual’s functioning at work [61].

In the organizational category, similar to the present findings, previous research on working women in Bangladesh showed that long working hours, insufficient organizational support, inappropriate policies and high workload cause conflict. Although they cited the lack of family support as an important issue for women, consistent with the present findings, they noted that it is not as widespread as work issues [13]. In addition, Obago studied WLC among 250 working women in Nigeria and found that factors such as long working hours, busy work schedule, inadequate work facilities, and high workloads increase the level of conflict among women [62].

Cultural factors formed a separate category in this study. The higher WLC in women around the world may be due to the fact that, in most countries, women traditionally manage family duties and are primarily in charge of caring for children and the elderly [63].

Iranian culture is a family-centered one, in which there are more demands and expectations in families compared with individual-centered societies. Due to the traditional gender roles, women are mainly responsible for family duties and men are less involved in domestic chore such as looking after children. Therefore, women with family roles must also do their occupational duties without any reduced share of domestic duties. Furthermore, in family-centered communities, looking after the elderly is a duty of children and few families take the responsibility of taking the elderly to nursing homes. The elderly live either with their children or alone in their houses while their children care for them. Most of the elderly, especially women, do not get married after their husband’s death and their children look after them. Meeting these expectations can create time-bound conflict with work responsibilities [64].

In the present study, the consequences of conflict on different aspects of life included the 2 main categories of lowering the quality of life and work issues, with 4 subcategories of physical and mental diseases, forgetting other roles, limited social communication and reduced productivity. These will disturb a proper balance between work and rest, and causes a lack of control over workload and lack of energy to achieve goals and commitments. These, in turn, lead to fatigue, poor performance and reduced quality of life [65], and contribute to poor physical and mental health [66, 67] Still, some previous studies indicated similar poor health and WLC in male and female employees [68].while others observed it more in female employees than in males [69, 70].

Conflict resolution methods include 3 main categories of individual-oriented methods, other-oriented methods, and organization-oriented methods with 8 sub-categories of program-oriented, meaning-oriented, emotion-oriented, avoidance, emotional support, instrumental and work support, support work policies, and correct management views. The present findings showed that the participants expected their managers to have the right managerial perspectives to manage tasks and workloads. As for WLC solutions in women, McGinnity and Russell pinpointed that flexible work arrangements such as part-time work are essential methods for WLB. Contrary to the present research [71], these researchers emphasized that working at home is associated with a significant workload and WLC. The present research found that women consider telecommuting as a suitable solution for WLC.

Kamrani et al., who studied 225 male and female nurses in six hospitals, showed that job flexibility, job commitment, and work support had the greatest impact on WLB [72]. Therefore, as expected, these factors should be considered for all employees regardless of their gender.

WLC and WLB are theoretically and practically essential for workers and organizations. A higher WLC may decrease satisfaction and performance, and may increase employee turnover, stress, intention to leave and absence from work [73],Since it has high and destructive financial costs for the organization, training new employees is important to be effective in their jobs. As a result, there are consequences such as alternative employment for workers. Organizations have to consider ways to reduce possible leave due to WLC [74]. The present participants mentioned supportive accommodations, part-time work, incentive leave, performance-dependent payment, reduced hours, and telecommuting as the possible solutions.

It is hypothesized that the level of WLC is related to demands and resources at home. The present study has recurrently raised the importance of child care and showed that finding a solution to this issue could significantly affect WLC. Mothers showed to have a higher level of WLC, and such policy measures as part-time job, flexibility and telecommuting can improve the quality of life Working at home has several direct positive effects, such as no need to commute, easier management of household responsibilities and family demands, along with increased independence during work time [75].

In this study, female employees were successfully interviewed to find important causes, effects and solutions to reduced WLC. However, there were some limitations too. A cross-gender comparison of these issues among employees can guide researchers and policy makers. Moreover, considering the different cultural beliefs in our country, it seems that more multicenter studies can be useful in other parts of the country.

## Conclusion

In the light of the present findings, it can be concluded that in addition to providing individual solutions such as learning skills, determining appropriate work policies that can help women manage family responsibilities alongside their work need to be seriously considered. The support of the surrounding people, especially the husband, along with supportive and family-friendly organizational policies can reduce WLC and should be taken into account.

### Supplementary Information

**Additional file 1.**

## Data Availability

All information extracted or analyzed during this study is included in this published article without mentioning the identity of the subjects.

## Abbreviations

WLB: Work-life balance
WLC: Work-life conflict
