# Supplementary material for: Etiology, consequences, and solutions of working women’s work-life conflict: a qualitative study
Source: BMC Womens Health. 2024 Jan 23;24:62. doi: 10.1186/s12905-023-02873-4 (PMC10804774; doi:10.1186/s12905-023-02873-4)
Supplement: Supplementary file 1 — Additional file 1. [file 12905_2023_2873_MOESM1_ESM.docx]

**Semi-structured questionnaire**

- What factors cause conflict between your work and life?

- What is your first reaction in a conflict situation?

- What methods do you have to control conflict situations?

- What things can help to solve the problem of conflict between work and life?

- What is the impact of conflicts between work and life and the resulting conflict on your work and family life?
